# Effect of Ethanol/Water Solvents on Phenolic Profiles and Antioxidant Properties of Beijing Propolis Extracts

**DOI:** 10.1155/2015/595393

**Published:** 2015-08-17

**Authors:** Chunli Sun, Zhengshuang Wu, Ziyan Wang, Hongcheng Zhang

**Affiliations:** ^1^Institute of Apicultural Research, Chinese Academy of Agricultural Sciences, Beijing 100093, China; ^2^College of Food Science and Technology, Nanjing Agricultural University, Nanjing 210095, China; ^3^National Research Center of Bee Product Processing, Ministry of Agriculture, Beijing 100093, China

## Abstract

Propolis is a natural substance known to be beneficial for human health and used as a folk medicine in many parts of the world. In this study, phenolic profiles and antioxidant properties of Beijing propolis extracted by different ethanol/water solvents were analyzed. Our results reveal that phenolic compounds and antioxidant properties of propolis extracts were significantly dependent on the concentration of ethanol/water solvents. Totally, 29 phenolic compounds were identified: 12 phenolic acids, 13 flavonoids, and 4 phenolic acid esters. In particular, 75 wt.% ethanol/water solvent may be the best for the highest extraction yield and the strongest antioxidant properties. Caffeic acid, benzyl caffeate, phenethyl caffeate, 5-methoxy pinobanksin, pinobanksin, pinocembrin, pinobanksin-3-O-acetate, chrysin, and galangin were the characteristic compounds of Beijing propolis, and these compounds seem to verify that Beijing propolis may be poplar-type propolis. In addition, the presence of high level of pinobanksin-3-O-acetate in Chinese propolis may be a novel finding, representing one-third of all phenolics.

## 1. Introduction

Propolis is a resinous substance collected by* Apis mellifera* L. from buds and exudates of different plant sources. It is also mixed with beeswax, pollen, and some certain enzymes from bees' saliva [1]. The chemical composition of propolis is diverse and complex. Approximately, 300 compounds have been identified from propolis, including flavonoids, phenolic acids, terpenoids, steroids, and amino acids [2]. Propolis has been used as a traditional medicine for thousands of years; thus, it has been extensively investigated in many application fields [2, 3]. Propolis covers a broad spectrum of biological effects from anticancer [4] and antioxidant [5] to antiviral [6] and anti-inflammatory [7] properties. These biological properties can mainly be ascribed to phenolic compounds, in particular phenolic acids and flavonoids.

Generally, phenolics of propolis are prepared with solvent extraction method [5, 8–10]. The advantages of the method are simple operation and low energy consumption. Among extraction solvents, nonpolar organic solvents mainly include ethyl acetate, chloroform, and n-butanol and polar solvents water, methanol, and ethanol.

Many studies have focused on the constituents and antioxidant activities of propolis extracts with different extraction solvents [5]. In previous research, compared to ethanol extracts, chloroform, n-butanol, and ethyl acetate extracts of propolis contained the higher content of total phenolics and exhibited the strongest antioxidant activity [5]. Similarly, ethanol extracts of propolis (EEP) showed a lower total phenolic content and a weaker antioxidant activity [8]. According to Laskar et al. [9], water extracts of propolis (WEP) with a higher phenolic content also illustrated a higher reducing power and radical-scavenging activity than EEP. On the contrary, other studies revealed that ethanol/water solvents were more effective in extracting phenolic compounds than water, and the ethanol extracts exhibited a higher antioxidant activity than aqueous extracts [10]. Thus, these inconsistent results seem to imply that different extraction solvents can affect phenolic composition and antioxidant properties of propolis extracts. However, to our knowledge, few studies focus on phenolic profiles and antioxidant properties of propolis extracts by using different ethanol/water solvents.

The purpose of this paper is to investigate phenolic profiles and antioxidant properties of Beijing propolis extracts by using different ethanol/water solvents. We analyzed phenolic composition of propolis extracts by high-performance liquid chromatography with PDA detection (HPLC-PDA). Moreover, we used five assays to evaluate antioxidant activities of propolis extracts: 1,1-diphenyl-2-picrylhydrazyl (DPPH) radical-scavenging activity, 2,2-azinobis (3-ethylbenzothiazoline-6-sulfonic acids) (ABTS) radical-scavenging activity, ferric reducing/antioxidant power assay (FRAP), oxygen radical absorbance capacity (ORAC), and cell antioxidant activity (CAA).

## 2. Materials and Methods

### 2.1. Chemicals

3,4-Dihydroxybenzaldehyde, vanillic acid, caffeic acid,* p*-coumaric acid, ferulic acid, isoferulic acid, benzoic acid, 3,4-dimethoxycinnamic acid, cinnamic acid, 4-methoxycinnamic acid, phenethyl caffeate, cinnamyl cinnamate, pinobanksin, quercetin, alpinetin, kaempferol, apigenin, isorhamnetin, pinocembrin, chrysin, galangin, 2′,7′-dichlorodihydrofluorescein diacetate (DCFH-DA), dimethyl sulfoxide (DMSO), Dulbecco's modified eagle medium (DMEM), Hanks's Balanced Salt Solution (HBSS), and fetal bovine serum (FBS) were from Solarbio Science & Technology Co., Ltd. (Beijing, China); DPPH (1,1-diphenyl-2-picrylhydrazyl), sodium fluorescein, ABAP (2,2-azobis (2-amidinopropane) dihydrochloride), Trolox (6-hydroxy-2,5,7,8-tetramethylchroman carboxylic acid), and Folin-Ciocalteu phenol reagent were purchased from Sigma-Aldrich Chemical Co., Ltd. (St. Louis, MO, USA); Cinnamylideneacetic acid and benzyl caffeate were from Funakoshi Company (Tokyo, Japan). Pinobanksin-3-O-acetate, pinostrobin, and tectochrysin were got from BioBioPha Co., Ltd. (Kunming, China); benzyl* p-*coumarate and 5-methoxy pinobanksin were prepared by ourselves.

Ethanol (analytical grade) and methanol (HPLC grade) were purchased from Fisher Scientific (Fair Lawn, NJ). Acetic acid (HPLC grade) was obtained from J. T. Baker (Phillipsburg, NJ). Water used to prepare the solutions or mobile phase was purified by a Milli-Q-Integral System (Merk Millipore, MA, USA). Human hepatocellular carcinoma (HepG2) cells were purchased from the Type Culture Collection of the Chinese Academy of Science (Shanghai, China).

### 2.2. Propolis Samples

Raw propolis was directly collected from hives of* Apis mellifera* L. located in the apiary of Bee Research Institute of Chinese Academy of Agricultural Sciences, Beijing Botanical Garden (Beijing, China). Raw propolis sample was frozen at −18°C for 4 hours and then crushed into homogeneous powder in a pulverizer (FW135, Tianjin, China). The powder sample was stored at −18°C until used.

### 2.3. Extraction

The propolis powder (1 g) was added to different extraction solvents (20 mL), such as water, 25, 50, 75, 95, and 100 wt.% ethanol/water solvents, respectively. The mixtures were treated by ultrasound for 5 h (100 W, 40°C). Subsequently, they were centrifuged at 400 ×g for 5 min (SORVALL Stratos 208 V, Thermo Fisher Scientific, USA).

The supernatants (5 mL) were used to measure the total phenolic content (TPC) and the total flavonoid content (TFC) as well as phenolic composition and antioxidant properties of propolis. On the other hand, the supernatants (10 mL) were concentrated with rotary evaporator (Buchi Co., Ltd., Rotavapor R-215, G4B21972A3704B, Switzerland). Then, the dried extracts were used to determine the extraction yield of propolis. Water extracts of propolis were expressed as WEP. Extracts of propolis by using 25, 50, 75, 95, and 100 wt.% ethanol/water solvents were expressed as 25% EEP, 50% EEP, 75% EEP, 95% EEP, and 100% EEP, respectively.

### 2.4. Extraction Yields of Propolis

The dried extracts were weighed to obtain the extraction yields from the following equation: Extraction yield (%) = (*W*_dryness_/*W*_propolis_). *W*_dryness_ is represented as the weight of the dry extracts; *W*_propolis_ is represented as the weight of raw propolis.

### 2.5. Total Phenolic and Flavonoid Contents

Total phenolic content (TPC) of propolis extracts was determined according to Folin-Ciocalteu method with slight modifications [13]. Every extraction solution (0.5 mL) of propolis was mixed with 2 N 0.5 mL of Folin-Ciocalteu reagent for 6 min. After the addition of 1.5 mL 20% Na_2_CO_3_, the volume was made up to 10 mL with corresponding extraction solvents, followed by incubation for 10 min at room temperature. Absorbance of the mixture was measured at 765 nm using UV-2500/Ultraviolet visible spectrophotometer (Shimadzu Co., Ltd., Tokyo, Japan). TPC was calculated from the calibration curve of gallic acid (*Y* = 0.0819*X* − 0.0071; *R* ^2^ = 0.9991) and expressed as milligram of gallic acid equivalent per gram of propolis (mg GAE g^−1^).

Total flavonoid content (TFC) was determined by a colorimetric method using aluminum chloride [14]. Every extraction solution of propolis (0.5 mL) was mixed with 0.3 mL of 5% NaNO_2_. After incubating for 6 min, 0.3 mL of 10% Al(NO_3_)_3_ was added. Following incubation of 6 min, 4 mL of 4.3% NaOH was added. Then the mixture was diluted with corresponding extraction solvents to 10 mL. After incubation for 15 min at room temperature, the absorbance was measured at 510 nm using UV-2500/Ultraviolet visible spectrophotometer (Shimadzu Co., Ltd., Tokyo, Japan). TFC was calculated from the calibration curve of rutin (*Y* = 0.0124*X* − 0.0002; *R* ^2^ = 0.9989) and expressed as milligram of rutin equivalent per gram of propolis (mg RE g^−1^).

### 2.6. Phenolic Composition Analysis with HPLC-PDA-MS

To identify the phenolic composition of propolis extracts, we used HPLC system with PDA detection (PDA-20A diode array detector, SIL auto-injection valve, CTO-10A thermostat, and pump LC-6AD, Shimadzu, Tokyo, Japan), coupled with a quadrupole time-of-flight mass spectrometer (Q-TOF MS) (Agilent 6540, Agilent Technologies, USA). Propolis extracts (10 *μ*L) were injected into the HPLC system equipped with a reversed-phase column Gemini C18 (150 × 4.6 mm, 5 *μ*m) (Phenomenex, Inc., CA, USA). The mobile phase consisted of 2% acetic acid in water (A) and 2% acetic acid in methanol (B) with a constant solvent flow rate of 0.75 mL/min. A 150 min linear gradient was programmed as follows: 0–25 min, 22%–36% B; 25–55 min, 36–52% B; 55–90 min, 55–63% B; 90–115 min, 63–70% B; 115–135 min, 70–75% B; 135–150 min, 75–80% B. The operating parameters of MS were as follows: source voltage, 4 kV; capillary voltage, 130 V; capillary temperature, 350°C. All MS data were acquired in the positive ionization mode. Twenty-nine phenolic compounds of propolis extracts were quantified at a wavelength of 280 nm by using external calibration curves.

Validation was carried out determining the limit of detection (LOD) and limit of quantitation (LOQ), as well as repeatability and reproducibility. Calibration curves of 29 phenolic compounds were between 0.991 and 0.999. The LODs of 29 phenolic compounds ranged from 0.02 *μ*g/kg to 8.79 *μ*g/kg. The LOQs ranged from 0.07 *μ*g/kg to 29.29 *μ*g/kg.

### 2.7. DPPH Radical-Scavenging Activity

DPPH radical-scavenging activity was measured according to the method [15] with slight modifications. Briefly, 200 *μ*L of various concentrations of propolis extracts (0.1–1.0 mg/mL) and 1.8 mL of corresponding extraction solvents were added to 2 mL of 0.2 mM ethanol solution of DPPH. The resulting solution was thoroughly mixed and incubated in the dark for 20 min at room temperature. Absorbance of the solution was measured at 517 nm using UV-2500/Ultraviolet visible spectrophotometer (Shimadzu Co., Ltd., Tokyo, Japan). The blank sample only contained the same volume of corresponding extraction solvents. Trolox was used as antioxidant standard. DPPH radical-scavenging activity was expressed as IC_50_ (concentration of total phenolics able to scavenger 50% of DPPH free radical).

### 2.8. ABTS Radical-Scavenging Activity

The ABTS radical-scavenging activity was measured as performed by Re et al. with slight modifications [16]. Firstly, stock solution of ABTS (7 mM) was mixed with potassium persulfate (140 mM) solution, and the ABTS radical cation was produced by adding potassium persulfate to a final concentration of 2.45 mM. Then, the ABTS solution was kept in the dark for 16 h at room temperature. Before analysis, the solution was diluted with anhydrous ethanol to an absorbance of 0.7 (±0.02) at 734 nm. All measurements were performed as follows: 100 *μ*L propolis extracts of various concentration (0.1–1 mg/mL) were mixed with 3 mL ABTS radical solution. Then the mixture was vibrated for 30 seconds, followed by standing for 6 min. The absorbance was measured at 734 nm using the same volume of corresponding extraction solvents as the blank sample. Trolox was used as antioxidant standard and evaluated at different concentrations.

### 2.9. Ferric Reducing/Antioxidant Power Assay

Ferric reducing/antioxidant power (FRAP) assay was carried out according to the method described by Benzie and Strain with slight modifications [17]. Briefly, 200 *μ*L propolis extracts of various concentrations (0.1–1 mg/mL) were mixed with 2.5 mL PBS buffer (0.2 M, pH 6.6) and 2.5 mL potassium ferricyanide (1.0%). Then the mixture was incubated for 20 min at 50°C. After 1.0 mL 10% trichloroacetic acid was added, the mixture was centrifuged at 2500 ×g for 10 min. Then 2.5 mL supernatant was mixed with 2.5 mL distilled water and 0.5 mL 0.1% ferric chloride. Absorbance of the final mixture was measured at 700 nm using UV-2500/Ultraviolet visible spectrophotometer (Shimadzu Co., Ltd., Tokyo, Japan). Aqueous solutions of Trolox concentrations in the range of 100 to 200 *μ*g/mL were used for the calibration, and the FRAP values were expressed as microgram of Trolox equivalents per milligram of propolis (mg Trolox/mg).

### 2.10. ORAC Assay

The ORAC assay was carried out as described by Prior et al. with minor modifications [18]. A precision 8-channel pipette was used for the transfer of solution. A Synergy TM HT microplate reader (BioTek Instruments Inc., USA) was used with fluorescence filters for an excitation wavelength of 485 nm and an emission wavelength of 528 nm. The final assay solution contained 150 *μ*L of 8.16 × 10^−2^ *μ*M fluorescein working solution, 20 *μ*L of phosphate buffer (blank), Trolox standard, or propolis extracts. The mixture was preincubated at 37°C for 10 min before 30 *μ*L of prepared ABAP (153 mM) was added. The fluorescence of the mixture solution was recorded every minute for a total of 50 min. The ORAC values were expressed as micromoles of Trolox equivalents per 100 g propolis (*μ*mol Trolox/100 g).

### 2.11. Cell Antioxidant Assay

The CAA assay was performed by using the method with slight modifications [19]. Human hepatocellular carcinoma HepG2 cells were seeded with a density of 1 × 10^6^/mL on a 96-well microplate in 100 *μ*L of growth medium per well. Twenty-four hours after seeding, the growth medium was removed and the wells were washed by PBS. Then 25 *μ*M DCFH-DA dissolved in treatment medium without FBS and 100 *μ*L propolis extracts of different concentration (0.25–0.5 mg/mL) were added to those wells and incubated at 37°C and 5% CO_2_ for 50 min. The wells were then washed with precooling PBS three times. After all these procedures, 600 *μ*M/L ABAP was added to the wells. Finally, fluorescence decay (*λ*_ex_ = 485 nm, *λ*_em_ = 538 nm) was monitored every 5 min for a period of 1 h in a Synergy TM HT microplate reader (BioTek Instruments Inc., USA). Each plate included triplicate control and blank wells: control wells contained cells treated only with DCFH-DA and ABAP; blank wells contained cells treated with dye and HBSS without ABAP. After blank subtraction from the fluorescence readings, the area under the fluorescence versus time curve was integrated to calculate the CAA value of propolis extracts as follows:

(1) $$\begin{array}{c} \text{CAA unit}=100-\left(\frac{\int SA}{\int CA}\right)\times 100, \end{array}$$

where ∫SA is the integrated area under the sample fluorescence versus time curve and ∫CA is the integrated area from the control curve. The CAA was represented as IC_50_.

### 2.12. Statistics

Analyses were carried out in triplicate and these data were expressed as means ± standard deviation (SD). One-way analysis of variance (ANOVA) followed by Duncan's Multiple-Range Test was used to compare significant differences among the different antioxidant activities. Statistical analyses were performed by using SAS version 9.1 (SAS Institute Inc., Cary, NC, USA). Significant differences were statistically considered at the level of *p* < 0.05.

## 3. Results

### 3.1. Extraction Yields, Total Phenolic, and Flavonoid Contents of Propolis

Phenolic compounds of propolis can play a protective role against oxidative damage caused by free radicals. In particular, flavonoids have been described as the main group of phenolic compounds responsible for biological properties.  Table 2 shows the extraction yields, TPC, and TFC treated by different ethanol/water solvents. The results presented that extraction yields ranged from 1.8% to 51% and tended to increase with increasing of the ethanol concentration.

TPC of various propolis extracts distinctly varied and ranged from 6.68 to 164.20 mg GAE g^−1^. Compared with TPC, TFC ranged from 4.07 to 282.83 mg RE g^−1^. In addition, the highest TPC and TFC were both observed in 75% EEP, followed by those in 95% EEP and 100% EEP, being the lowest in the WEP.

### 3.2. Phenolic Compositions of Propolis Extracts

**Figure 1:**
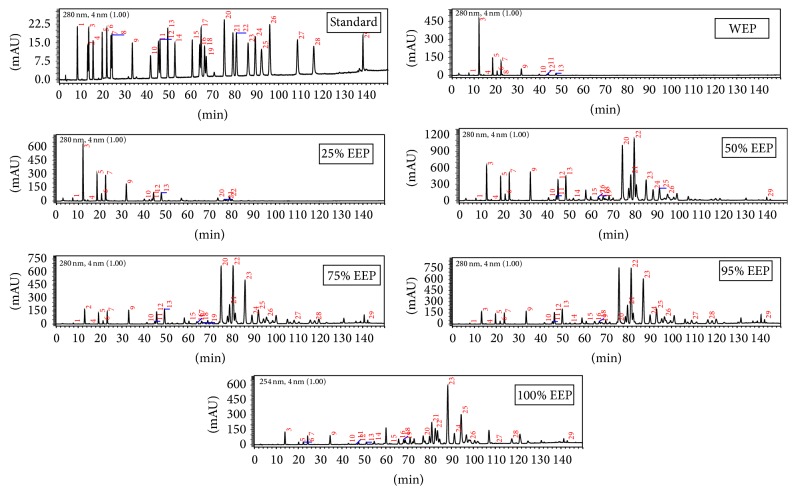
HPLC chromatograms of different EEP (recorded at 280nm). (1) 3,4-Dihydroxybenzaldehyde, (2) vanillic acid, (3) caffeic acid, (4)* p-*coumaric acid, (5) ferulic acid, (6) isoferulic acid, (7) benzoic acid, (8) 3,4-dimethoxycinnamic acid, (9) cinnamic acid, (10) 4-methoxycinnamic acid, (11) cinnamylideneacetic acid, (12) 5-methoxy pinobanksin, (13) pinobanksin, (14) quercetin, (15) alpinetin, (16) kaempferol, (17) cinnamylideneacetic acid, (18) apigenin, (19) isorhamnetin, (20) pinocembrin, (21) benzyl caffeate, (22) pinobanksin-3-O-acetate, (23) chrysin, (24) phenethyl caffeate, (25) galangin, (26) benzyl* p-*coumarate, (27) pinostrobin, (28) tectochrysin, and (29) cinnamyl cinnamate.

Phenolic composition of propolis extracts was identified by HPLC-PDA-MS.  Figure 1 shows the HPLC profiles of propolis extracts by using different ethanol/water solvents. According to the HPLC-PDA profiles of propolis extracts and UV spectra of major peaks, these extracts can be classified into two types. The first type, including WEP and 25% EEP, exhibited simple chromatograms with limited amount of peaks which mainly located in the region of retention times up to 50 min. The second type, including 50, 75, 95, and 100% EEP, presented the complicated chromatograms. HPLC profiles of these extracts were extremely complex with many peaks between the retention times of 10 and 140 min.

Phenolic composition of all propolis extracts is shown in Tables 1 and 3. These phenolic compounds were identified by HPLC-PDA-MS and confirmed by previous spectrometry mass references [11, 12]. We identified 29 phenolics from propolis extracts, including 12 phenolic acids, 4 phenolic acid esters, and 13 flavonoids. A large number of phenolic compounds were contained ranging from 15 compounds in WEP to 28 compounds in 75% EEP. Among the phenolics of the first type extracts, the main compounds were polar phenolic acids, such as caffeic acid,* p*-coumaric acid, isoferulic acid, and benzoic acid. In contrast, the second type contained not only polar phenolic acids but also a great percentage of weak-polar phenolics, such as some flavonoids and four phenolic acid esters. On the other hand, contents of phenolic compounds also showed distinct variety among propolis extracts with the different solvent/water solvents. For example, caffeic acid possessed the highest content of 2.13 mg/g in WEP. But the content was obviously lower than 3.74 mg/g in 75% EEP. Pinocembrin and pinobanksin-3-O-acetate were abundantly detected in 75% EEP with the average amounts of 40.39 mg/g and 69.36 mg/g; these amounts were almost 30 times that of WEP. Particularly, the highest content of flavonoids in WEP was limited, 0.67 mg/g of pinobanksin, while in 75% EEP the highest content was 69.36 mg/g, approximately 100 times that in WEP.

For Beijing propolis we investigated, 75% EEP contained comprehensive phenolics. The main phenolic acids were caffeic acid,* p*-coumaric acid, isoferulic acid, 3,4-dimethoxycinnamic acid, and benzoic acid. The largest amount of phenolic acids was caffeic acid with a content of 3.74 mg/g propolis. Benzyl caffeate and phenethyl caffeate were the representative phenolic acid esters with an amount of 20.34 mg/g and 10.51 mg/g. Flavonoids primarily included 5-methoxy pinobanksin, pinobanksin, pinocembrin, pinobanksin-3-O-acetate, chrysin, and galangin. Among these flavonoids, pinobanksin-3-O-acetate illustrated the highest content of 69.36 mg/g, accounting for 32% of all phenolics. In general, the characteristic compounds of Beijing propolis were caffeic acid, benzyl caffeate, phenethyl caffeate, 5-methoxy pinobanksin, pinobanksin, pinocembrin, pinobanksin-3-O-acetate, chrysin, and galangin. These characteristic compounds represented nearly 90% of total phenolics.

### 3.3. Antioxidant Properties

Oxidation is a very complex process with different mechanisms; therefore, no one single method exists completely to evaluate the antioxidant property. In this study, antioxidant properties of propolis extracts were determined through five assays.

#### 3.3.1. DPPH Radical-Scavenging Activity

We used the DPPH, a stable-free radical, to evaluate the antioxidant activity of propolis extracts. The results are expressed as IC_50_. The lower IC_50_ value indicates the stronger antioxidant activity. The IC_50_ values of different propolis extracts varied from 13798 *μ*g/mL to 633 *μ*g/mL (Table 4). Based on the IC_50_ values, propolis extracts can also divide into two types. The IC_50_ values of WEP and 25% EEP in the first type were approximately more than 10-fold higher than those of other EEP in the second type. 75% EEP especially exhibited the strongest DPPH radical-scavenging activity; its IC_50_ value was 633 *μ*g/mL, much lower than that of WEP.

#### 3.3.2. ABTS Radical-Scavenging Activity

It was observed that the ABTS radical-scavenging activity assay presented the similar results of DPPH assay. The IC_50_ values of the second type were approximately 5% of the WEP and 10% of 25% EEP. In addition, 75% EEP showed the lowest IC_50_ value, indicating the highest ABTS radical-scavenging activity.

#### 3.3.3. Ferric Reducing/Antioxidant Power

The FRAP assay is an electron transfer method based on the reduction of a ferric-tripyridyltriazine complex to its ferrous in the presence of antioxidants [20]. As can be seen in Table 4, we can also divide FRAP values of propolis extracts into two groups: group one with low values of approximate 20 *μ*g Trolox/mg, including WEP and 25% EEP, and another group with high values of about 200 *μ*g Trolox/mg, including 50, 75, 95, and 100% EEP. Specifically, the 75% EEP illustrated the highest value indicating the strongest reducing ability, and the value was almost 15-fold of that of WEP.

#### 3.3.4. ORAC Assay

Oxygen radical absorbance capacity is a relatively standardized method to measure antioxidant capacities in biological samples [21]. As shown in Table 4, all the propolis extracts had high ORAC values and varied widely ranging from 1383 to 275954 *μ*mol Trolox/100 g propolis. The 75% EEP especially had the highest ORAC value of 275954 *μ*mol Trolox/100 g propolis and the value was almost 200-fold that of WEP and significantly higher than those of other EEP (*p* < 0.05).

#### 3.3.5. Cell Antioxidant Activity

There is increasing evidence that production of reactive oxygen species is involved in various disorders and also is responsible for cellular damage. Cellular antioxidant assay of propolis extracts was investigated on HepG2 cells. Table 4 shows that the second type propolis samples presented higher cell antioxidant activity than the first type. Additionally, CAA of 75% EEP increased 150-fold compared to WEP, indicating the highest CAA.

## 4. Discussions

Methanol, hexane, acetone, ethyl acetate, ethanol, and water are the common solvents to extract phenolic compounds of propolis, especially ethanol and water [2, 22]. Our studies aimed at investigating the effect of different ethanol/water solvents on the phenolic compositions and antioxidant properties of propolis extracts.

We evaluated the effect of different ethanol/water solvents on the yields of propolis extracts (Table 2). The results showed that WEP obtained the lowest yield, while extracts with ethanol/water solvents got higher yields, showing a great increase with ethanol concentrations. In addition, the highest TPC and TFC were both observed in 75% EEP (Table 2). Ethanol/water concentrations were highly correlated with extraction yields, TPC, and TFC (*r* = 0.86, 0.79, and 0.83, resp.). Thus, according to the results, water and 25 wt.% ethanol/water solvent seemed to be less effective in extracting phenolics than those ethanol/water extraction solvents with high concentrations. Our results are consistent with the previous studies. For example, Miyataka et al. [23] reported extraction yield with 99.5 wt.% ethanol to extract Brazilian propolis was 41–60%, in comparison with 4–14% of distilled water. Therefore, our results suggest that 75 wt.% ethanol/water solvent can be the best extraction solvent for phenolics in propolis.

Phenolic composition of propolis extracts using different ethanol/water solvents greatly varied, ranging from 15 compounds in WEP to 28 compounds in 75% EEP (Table 3). In the first type, phenolic acids accounted for about 60% of the total phenolic compounds. Such several flavonoids as pinobanksin, pinocembrin, and kaempferol in WEP were detected with low content. By contrast, the second type extracts contained rich variety of phenolics; flavonoids and phenolic acid esters especially were the main compounds occupying at least about 80% of total phenolics. Our results are consistent with the previous study, revealing that the constituents of WEP mainly were caffeic acid,* p*-coumaric acid, ferulic acid, and 3,4-dimethoxycinnamic acid [12]. In addition, it was worthwhile to note in our study that 75% EEP contained rich variety of phenolics, not only polar compounds but also other weak-polar and apolar compounds. Similar results were illustrated in the previous research, reporting that ethanol/water solvent is particularly suitable to obtain propolis extracts rich in phenolic components, especially flavonoids with high contents [1]. This may be due to the fact that aqueous solvents are suitable for extracting some bioactive compounds with strong polarity; ethanol or ethanol/water solvent is suitable for extracting some bioactive compounds with broad range of polarity. Thus, these results imply that 75 wt.% ethanol/water solvent may be appropriate to extract phenolics of propolis.

Antioxidant properties of propolis extracts were determined through different methods including DPPH, ABTS, FRAP, ORAC, and CAA methods. Our study revealed that these propolis extracts with complex phenolic composition presented higher antioxidant activities than the propolis extracts with lower content of phenolics (Tables 1 and 3). The EEP illustrated stronger antioxidant activity than WEP; in particular, 75% EEP possessed the strongest antioxidant activities. Similar results were observed by Mello and Hubinger [24], reporting that the ethanol extracts exhibited higher antioxidant activity than that of aqueous extracts. Another study also presented that the ethanol/water solvent could affect the antioxidant activity of propolis extracts [20]. According to our ORAC data, propolis possesses the strongest antioxidant activity, compared to other foods in USDA Database for the Oxygen Radical Absorbance Capacity (ORAC) [25, 26].

Our study showed that for 75% EEP, caffeic acid, benzyl caffeate, phenethyl caffeate, 5-methoxy pinobanksin, pinobanksin, pinocembrin, pinobanksin-3-O-acetate, chrysin, and galangin were the characteristic compounds (Table 3). From the characteristic compounds, we may speculate that Beijing propolis probably originate in the phenolic resins which mainly contain phenolics, such as lipophilic flavonoids and some esters. Moreover, these characteristic compounds of propolis extracts are similar to those of phenolic resin from poplar buds [27]. The previous studies have demonstrated that characteristic compounds of the propolis from China are pinocembrin, chrysin, galangin, and tectochrysin and the source plant is* Populus* spp. (poplar) [2, 23]. Thus, our study appears to verify that Beijing propolis may be poplar-type propolis. In addition, our result showed that pinobanksin-3-O-acetate possessed the highest content, representing one-third of all phenolics. However, previous studies presented that the content of chrysin was the highest among all phenolic compounds in Chinese propolis [28–30]. Thus, the presence of such high level of pinobanksin-3-O-acetate in Chinese propolis may be a novel finding. It should be noted that this study was primarily concerned with the Beijing propolis. The lack of enough propolis samples can mean that our finding needs to be explored in future work.

Some authors have showed that antioxidant activity was principally due to the total phenolics [31] or some key phenolics [32]. To explore the influence of phenolics on antioxidant activity, the correlation between the phenolic contents and antioxidant activity was determined. The antioxidant activity of propolis extracts appears to be largely influenced by the TPC and TFC. Positive correlation is observed between TPC and all antioxidant activities (*r* = 0.70–0.87) and between TFC and all antioxidant activities (*r* = 0.76–0.85). This observation is in agreement with the previous research reporting a positive correlation between antioxidant activities and TPC [5]. Since there were a large number of antioxidant phenolics in the propolis (Table 3), it was not clear which compounds were responsible for the antioxidant activity. In this study we evaluated the relationship between the characteristic constituents and the antioxidant activities. Our results revealed that benzyl caffeate and phenethyl caffeate were related to oxygen radical absorption capacity in ORAC assay (*r* = 0.74, 0.75). Such flavonoids as 5-methoxy pinobanksin, pinobanksin, pinocembrin, pinobanksin-3-O-acetate, and chrysin were highly correlated with the antioxidant activity (*r* = 0.76–0.85). Similarly, these compounds strongly associated with CAA assay (*r* = 0.77–0.97). Corresponding results were illustrated in the previous research [32], showing that phenethyl caffeate and flavonoids present strong antioxidant activity. Therefore, the antioxidant activity of propolis could be related to the presence of specific blends of phenolic compounds.

## 5. Conclusions

Ethanol/water solvents present significant effect on the phenolic composition and antioxidant properties of propolis extracts. Moreover, 75 wt.% ethanol/water solvent might be suitable to extract phenolics of propolis. To our knowledge, this is the first study that concretely described effects of series of known concentrations of ethanol/water solvents on phenolic composition and antioxidant activities of Beijing propolis. Caffeic acid, benzyl caffeate, phenethyl caffeate, 5-methoxy pinobanksin, pinobanksin, pinocembrin, pinobanksin-3-O-acetate, chrysin, and galangin were the characteristic compounds of Beijing propolis. These compounds seem to verify that Beijing propolis may be poplar-type propolis. Therefore, further studies on plant origin of Chinese propolis are still needed.

## Figures and Tables

**Table 1:** Phenolics identified in propolis samples and their UV and MS characteristics.

	Phenolics	UV *λ*max (nm)	Mw	[M + H]^+^	[M + Na]^+^	Reference
1	3,4-Dihydroxybenzaldehyde	280, 311	138	139.0387	—	[11]
2	Vanillic acid	260, 292, 369	168	169.0489	—	[12]
3	Caffeic acid	322	180	181.0501	—	[11]
4	Vanillin	280, 309	152	153.0549	—	[12]
5	*p-*Coumaric acid	309	104	165.0548	187.0366	[11]
6	Ferulic acid	323	194	195.0655	217.0474	[11]
7	Isoferulic acid	303, 323	194	195.0657	217.0477	[11]
8	Benzoic acid	273	122	123.0437	—	[12]
9	3,4-Dimethoxycinnamic acid	321	208	209.0816	231.0638	[11]
10	Cinnamic acid	276	148	149.0234	—	[11]
11	4-Methoxycinnamic acid	230, 309	178	179.0700	201.0509	[12]
12	Cinnamylideneacetic acid	311	174	175.0758	—	[11]
13	Benzyl caffeate	232, 328	270	—	293.0792	[11]
14	Phenethyl caffeate	301, 328	284	285.1122	307.0948	[11]
15	Benzyl *p-*coumarate	232, 313	254	255.0660	277.0482	[11]
16	Cinnamyl cinnamate	328	296	—	319.0946	[11]
17	5-Methoxy pinobanksin	232, 287	286	287.092	309.0741	[11]
18	Pinobanksin	291	272	273.076	295.0585	[11]
19	Quercetin	292, 365	302	303.0502	325.032	[11]
20	Alpinetin	286	270	271.0972	293.0795	[11]
21	Kaempferol	266, 296, 366	286	287.0554	309.0375	[11]
22	Apigenin	267, 338	270	271.064	—	[11]
23	Isorhamnetin	293, 370	316	317.0665	339.0489	[11]
24	Pinocembrin	290	256	257.0816	—	[11]
25	Pinobanksin-3-O-acetate	294	314	315.0868	337.0688	[11]
26	Chrysin	268, 313	254	255.0663	277.0844	[11]
27	Galangin	264, 357	270	271.0955	—	[11]
28	Pinostrobin	289	270	271.0974	293.0787	[12]
29	Tectochrysin	268, 311	268	269.0817	291.0635	[12]

Note: —: not detected.

**Table 2:** Total phenolic and flavonoid contents of propolis extracts.

Samples	Extraction yield	Total polyphenol content	Total flavonoids content
	(%)	(mg GAE g^−1^)	(mg RE g^−1^)
WEP	1.81 ± 0.05^a^	6.68 ± 0.01^ab^	4.07 ± 0.01^a^
25% EEP	3.71 ± 0.14^b^	15.79 ± 0.02^a^	15.22 ± 0.01^c^
50% EEP	42.14 ± 0.51^c^	149.90 ± 0.13^ab^	204.29 ± 0.13^d^
75% EEP	47.60 ± 0.79^d^	164.20 ± 0.07^bc^	282.83 ± 0.26^e^
95% EEP	49.36 ± 0.65^d^	162.34 ± 0.12^c^	246.03 ± 0.31^d^
100% EEP	51.03 ± 0.16^d^	152.03 ± 0.11^d^	250.86 ± 0.21^d^

Note: water extracts of propolis were expressed as WEP. Extracts of propolis by using 25, 50, 75, 95, and 100 wt. % ethanol/water solvents were expressed as 25% EEP, 50% EEP, 75% EEP, 95% EEP, and 100% EEP, respectively. TPC was expressed as milligram of gallic acid equivalent per gram of propolis (mg GAE g^−1^). TFC was expressed as milligram of rutin equivalent per gram of propolis (mg RE g^−1^). Dates are mean ± standard deviation (*n* = 3). Values in the same column followed by the same lowercased letter are not significantly different by Duncan's Multiple-Range Test (*p* < 0.05).

**Table 3:** Quantitation of the constituents in different propolis extract.

Components	Content (mg/g of propolis)					
	WEP	25% EEP	50% EEP	75% EEP	95% EEP	100% EEP
3,4-Dihydroxybenzaldehyde	0.03	0.11	0.11	0.10	0.11	0.11
Vanillic acid	0.01	0.02	—	—	—	—
Caffeic acid	2.13	3.42	3.74	3.74	3.50	3.30
Vanillin	0.05	0.12	0.17	0.16	0.18	0.18
*p-*Coumaric acid	0.37	1.08	1.33	1.40	1.50	1.32
Ferulic acid	0.22	0.63	0.92	0.94	0.88	0.87
Isoferulic acid	0.46	1.66	2.20	2.08	2.60	2.42
Benzoic acid	0.54	1.09	1.08	1.06	—	—
3,4-Dimethoxycinnamic acid	0.22	0.81	1.75	1.60	2.00	1.84
Cinnamic acid	0.02	0.04	0.10	0.10	0.11	0.09
4-Methoxycinnamic acid	0.08	0.19	0.55	0.57	0.65	0.59
Cinnamylideneacetic acid	—	0.07	0.30	0.34	0.37	0.30
Benzyl caffeate	—	1.55	20.20	20.34	23.54	22.36
Phenethyl caffeate	—	0.37	10.49	10.51	12.33	11.30
Benzyl *p-*coumarate	—	—	4.13	4.60	5.28	5.36
Cinnamyl cinnamate	—	—	0.36	0.51	0.57	0.51
5-Methoxy pinobanksin	0.54	1.84	8.02	7.91	7.92	7.99
Pinobanksin	0.67	1.82	8.06	7.66	7.85	8.01
Quercetin	—	—	0.22	0.10	0.20	0.21
Alpinetin	—	0.09	1.13	1.15	1.08	1.04
Kaempferol	0.02	0.05	0.59	0.59	0.58	0.57
Apigenin	—	0.03	0.88	0.89	0.94	0.85
Isorhamnetin	—	0.23	3.59	3.63	2.90	3.03
Pinocembrin	0.11	1.39	37.42	40.39	38.25	38.69
Pinobanksin-3-O-acetate	—	2.35	66.93	69.36	67.67	69.12
Chrysin	—	0.39	20.03	23.90	25.33	23.66
Galangin	—	0.27	9.97	11.25	12.22	11.53
Pinostrobin	—	—	1.81	2.85	2.81	2.13
Tectochrysin	—	—	1.45	1.92	2.11	2.00

Note: —: not detected.

**Table 4:** Antioxidant properties of different propolis extracts.

Samples	DPPH	ABTS	FRAP	ORAC	CAA
	IC_50_ (*μ*g/mL)	IC_50_ (*μ*g/mL)	*μ*g Trolox/mg	*μ*mol Trolox/10 g	IC_50_ (*μ*g/mL)
Trolox	90 ± 2^a^	78 ± 1^a^	1000	400	
WEP	13798 ± 641^b^	10310 ± 477^b^	20 ± 3^a^	1385 ± 96^a^	25738 ± 2269^a^
25% EEP	7129 ± 1950^b^	5520 ± 181^c^	16 ± 0^b^	9180 ± 179^b^	9907 ± 904^b^
50% EEP	759 ± 28^c^	613 ± 24^de^	214 ± 9^c^	241893 ± 13908^c^	214 ± 29^e^
75% EEP	633 ± 27^c^	520 ± 13^d^	233 ± 11^d^	275954 ± 11336^d^	171 ± 18^e^
95% EEP	705 ± 21^e^	547 ± 14^ef^	192 ± 21^e^	143372 ± 12090^d^	192 ± 21^e^
100% EEP	695 ± 22^e^	556 ± 13^f^	194 ± 21^e^	174593 ± 13340^e^	243 ± 30^d^

Note: dates are mean ± standard deviation (*n* = 3). Values in the same column followed by the same lowercased letter are not significantly different by Duncan's Multiple-Range Test (*p* < 0.05). DPPH (1,1-diphenyl-2-picrylhydrazyl) and ABTS (2,2-azobis (2-amidinopropane) dihydrochloride) were both expressed as IC_50_ (concentration of total phenolics able to scavenger 50% of free radicals); ferric reducing/antioxidant power (FRAP) assay was expressed as microgram of Trolox equivalents per milligram of propolis (mg Trolox/mg); oxygen radical absorbance capacity (ORAC) was expressed as micromoles of Trolox equivalents per 100 g propolis (*μ*mol Trolox/100 g) and cell antioxidant activity (CAA) was expressed as IC_50_.
